# Supplementary material for: The Phosphatidylserine Receptor TIM-1 Enhances Authentic Chikungunya Virus Cell Entry
Source: Cells. 2021 Jul 20;10(7):1828. doi: 10.3390/cells10071828 (PMC8303237; doi:10.3390/cells10071828)
Supplement: Supplementary file 1 [file cells-10-01828-s001.zip › cells-1266429-supplementary.pdf]

cells

Supplementary material

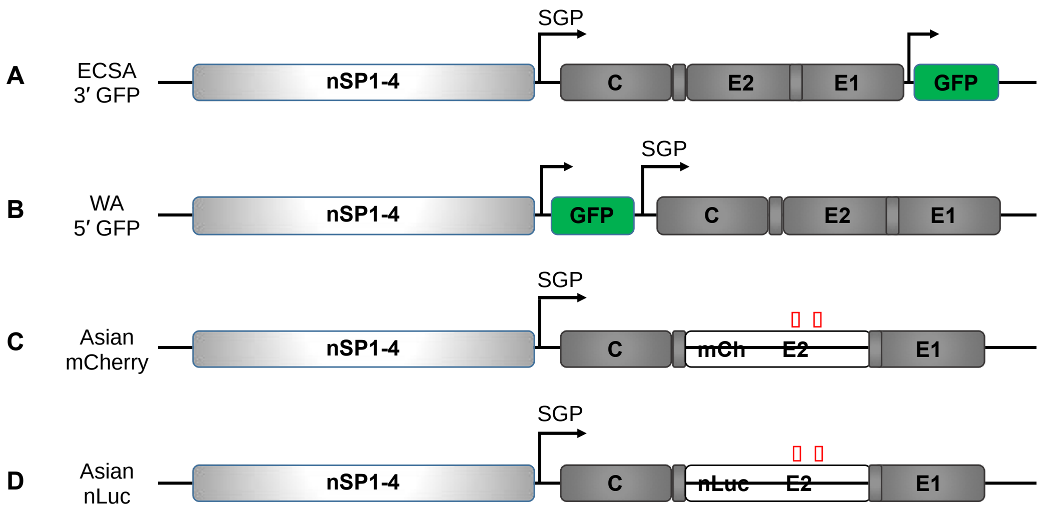

Figure S1. Schematic representation of CHIKV strains**.** (**A**) East Central South African (ECSA) strain encoding a green fluorescent protein (GFP) gene on the 3′ end of the genome (3′GFP CHIKV). (**B**) West African (WA) strain encoding a green fluorescent protein (GFP) gene 5′ end of the structural genes (5′GFP CHIKV). (**C**) Asian vaccine strain (181/25) encoding mCherry-fluorescent protein gene fused to the E2 gene hence the viral particles are fluorescent (mCherry-CHIKV). (**D**) Asian vaccine strain (181/25) encoding nano-luciferase gene fused to the E2 gene (nLuc-CHIKV). The Asian strain is attenuated due to amino acid substitutions at positions 12 and 82 in the E2 envelope glycoprotein (red asterisk).

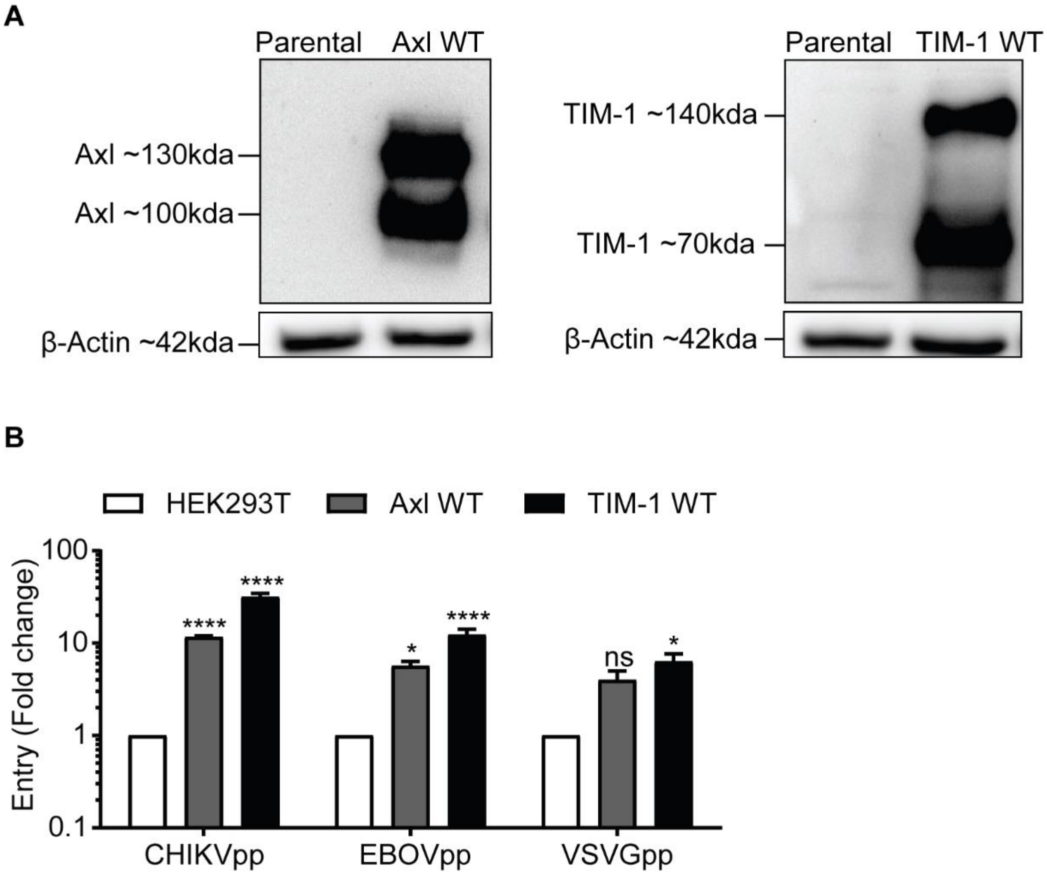

Figure S2: Expression of Axl and TIM-1 in HEK293T cells enhances entry of CHIKV glycoprotein-based pseudovirus. (**A**) Western blot of parental HEK293T cells and cells transduced to express Axl and TIM-1. Cells were selected for resistance to blasticidin and proteins in the cell lysate separated by SDS-PAGE. TIM-1 and Axl expression was probed with monoclonal antibodies. (**B**) Transduction of cells with lentiviral pseudoparticles encoding glycoproteins of CHIKV, EBOV and VSV to determine entry efficiency in parental HEK293T, Axl and TIM -1 expressing cells after 24h. VSVG pseudoparticles (VSVGpp) prediluted 1:100. Entry levels were assessed by luciferase assay. The error bars represent mean ± SEM of three independent experiments. Statistical significance was calculated using a Dunnet's multiple comparisons test (2way ANOVA) *p<0.05, **p<0.01, ***p<0.001 and ****p<0.0001.

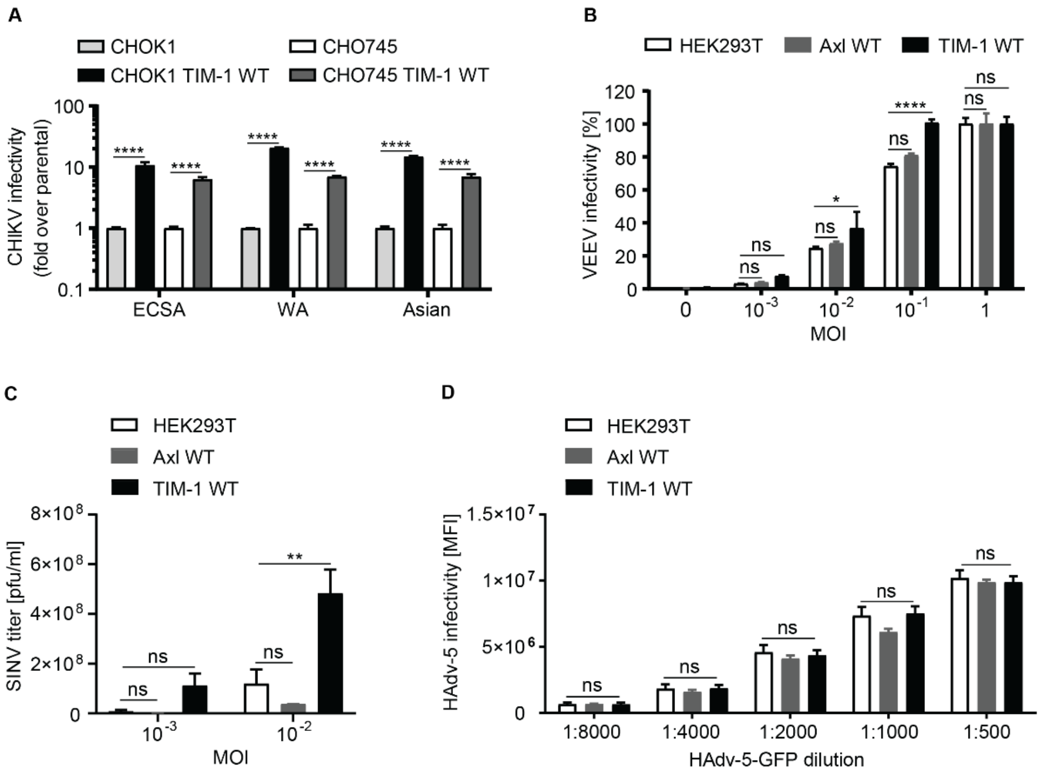

Figure S3: TIM-1-dependent infectivity of CHIKV genotypes and other *Alphavirus* species. (**A**) Parental and TIM-1 WT expressing CHO cells were inoculated with ECSA 3′GFP-CHIKV (MOI 0.01), 5′GFP-CHIKV WA (MOI 0.01) and Asian mc-CHIKV (MOI 0.1). At 24 hpi, infectivity was determined by flow cytometry. (**B**) Parental HEK293T, Axl WT and TIM-1 WT expressing cells were challenged with VEEV-GFP at indicated MOI. Percentage of infected cells were assessed by flow cytometry. (**C**) Parental HEK293T, Axl WT and TIM-1 WT expressing cells were challenged with SINV at indicated MOI. After 24h the supernatants were collected and titer taken on Vero cells. (**D**) Parental HEK293T, Axl WT and TIM-1 WT expressing cells were challenged with GFP expressing HAdV-5 at indicated dilutions. After 24 h the infectivity was determined by GFP expression imaged with a Trophos plate runner. The error bars represent mean ± SEM of three independent experiments. Statistical significance was calculated using a Dunnet's multiple comparisons test (2way ANOVA) *p<0.05, **p<0.01, ***p<0.001 and ****p<0.0001.

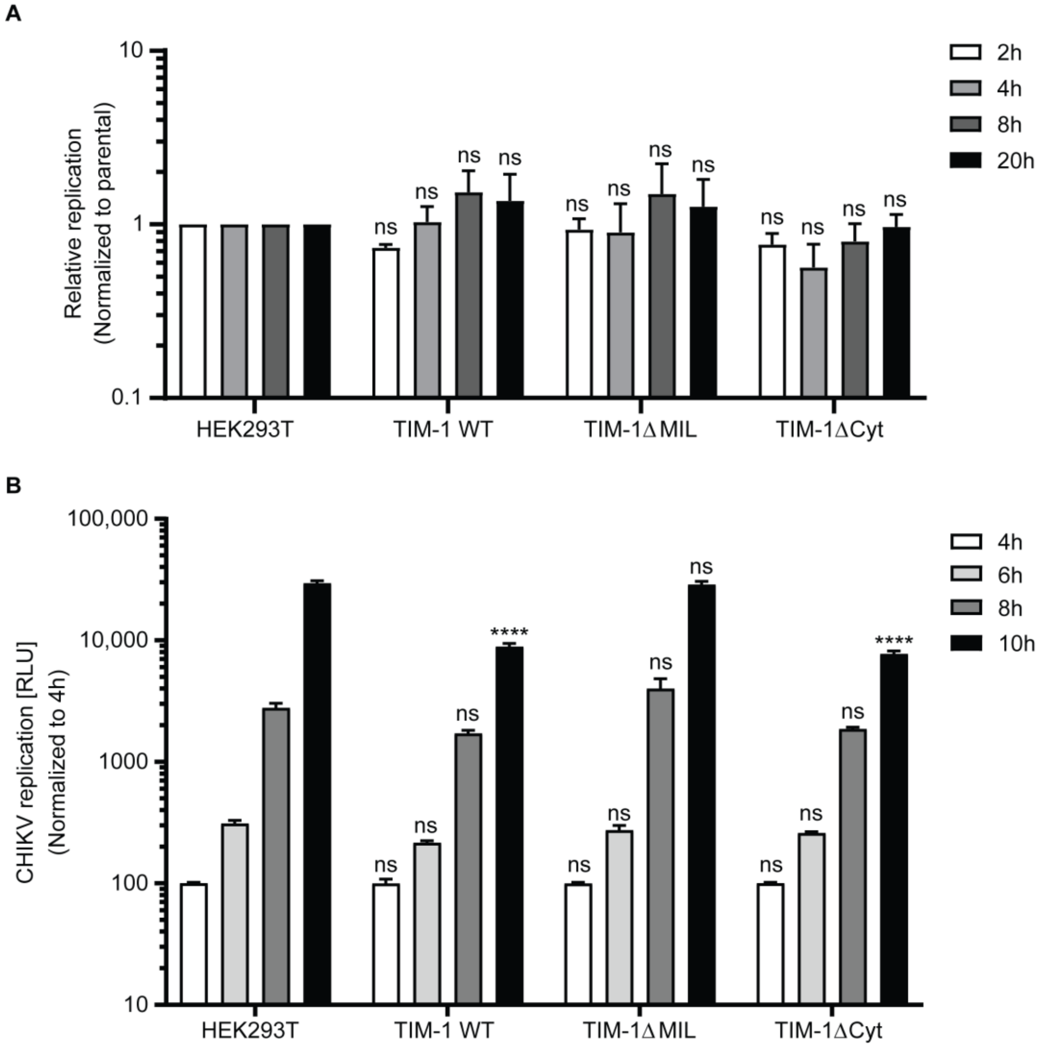

Figure S4: CHIKV replicates independently of TIM-1. (**A**) Parental HEK293T cells expressing TIM-1 WT, TIM-1ΔMIL and TIM-1ΔCyt were electroporated with CHIKV subgenomic RNA encoding luciferase gene. After 24 h, replication was determined using luciferase assay. (**B**) Parental HEK293T cells expressing TIM-1 WT, TIM-1ΔMIL and TIM-1ΔCyt were electroporated with authentic Asian CHIKV encoding nano-luciferase gene. After 10h, CHIKV replication was determined by luciferase assay. The error bars represent mean ± SEM of three independent experiments. Statistical significance was calculated using a Dunnet's multiple comparisons test (2way ANOVA) *p<0.05, **p<0.01, ***p<0.001 and ****p<0.0001.

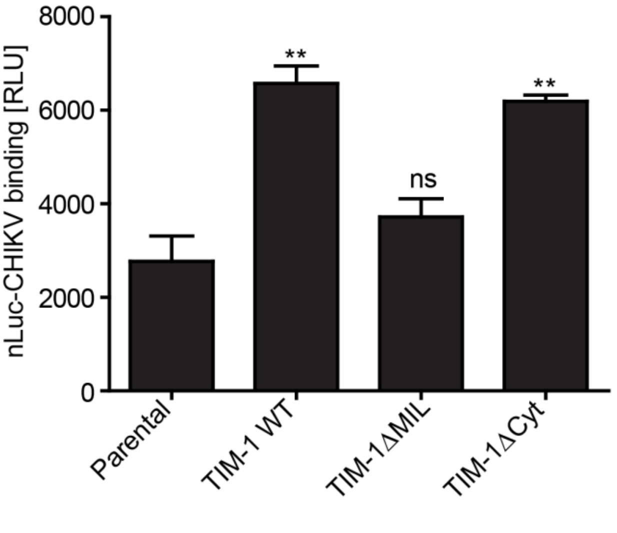

Figure S5: Relative binding of nLuc-CHIKV virus. Binding on the surface of parental and cells expressing TIM-1 WT, TIM-1ΔMIL and TIM-1ΔCyt. Cells were inoculated with Asian CHIKV encoding nano-luciferase gene and incubated at 4°C for 1 hour and binding detected by luciferase assay.

CHIKV Strain	ECSA			WA			Asian		
CHOK1 (% positive)	0.6	0.7	0.7	0.7	0.8	0.8	1.3	1.5	1.5
CHOK1-TIM1 WT (% positive)	6.2	6.6	8.9	16.0	15.4	15.4	19.7	22.0	22.0
CHO745 (% positive)	1.6	1.7	1.9	1.7	2.7	2.7	2.6	3.9	3.9
CHO745-TIM1 WT (% positive)	9.4	11.2	12.3	15.6	16.8	16.8	19.4	26.8	26.8

**Hours post infection**	**HaCat (nLuc-CHIKV, RLU)**			**TIM-1 WT (nLuc-CHIKV, RLU)**		
0	232	126	184	269.5	136.5	163.5
4	1889157	824547.5	768288	2635096	1045724	1093571
8	915185.5	718722.5	704849	1014474	712713	716341
12	1076710	425443	454877.5	1324928	933565	982788
24	516416.5	409974	456643	8131405	4972497	6395615
48	159200.8	147524.5	170825	3088506	3481855	3875003

**Hours post infection**	**TIM-1ΔMIL (nLuc-CHIKV, RLU)**			**TIM-1ΔCyt (nLuc-CHIKV, RLU)**		
0	297	116	129.5	238.5	112.5	116
4	2377758	638731	711947	2348411	558820	667805.5
8	1120652	731908.5	631266	1042929	635041	678559
12	850393.5	625406.5	639253.5	691587.5	481390.5	516043.5
24	710820.5	438678.5	469116.5	829659	610625.5	625040
48	231507.5	222521	240294	461672.5	487958.8	514165

**Hours post infection**	**Axl WT (nLuc-CHIKV, RLU)**		
0	228.5	102.5	109
4	1791784	458437	562135.5
8	963848.5	507544	576314.5
12	977991	469230.5	514147
24	800115.5	513822.5	527232.5
48	348055.8	263154	432935.5

Table S1: Baseline values after infection with authentic CHIKV. ECSA 3'GFP-CHIKV, 5'GFP-CHIKV WA and Asian mc-CHIKV used to infect CHO cells and percentage of infected cells are indicated. HaCat cells were infected with Asian CHIKV encoding nano-luciferase gene (nLuc-CHIKV) and relative light units indicate the infection levels.

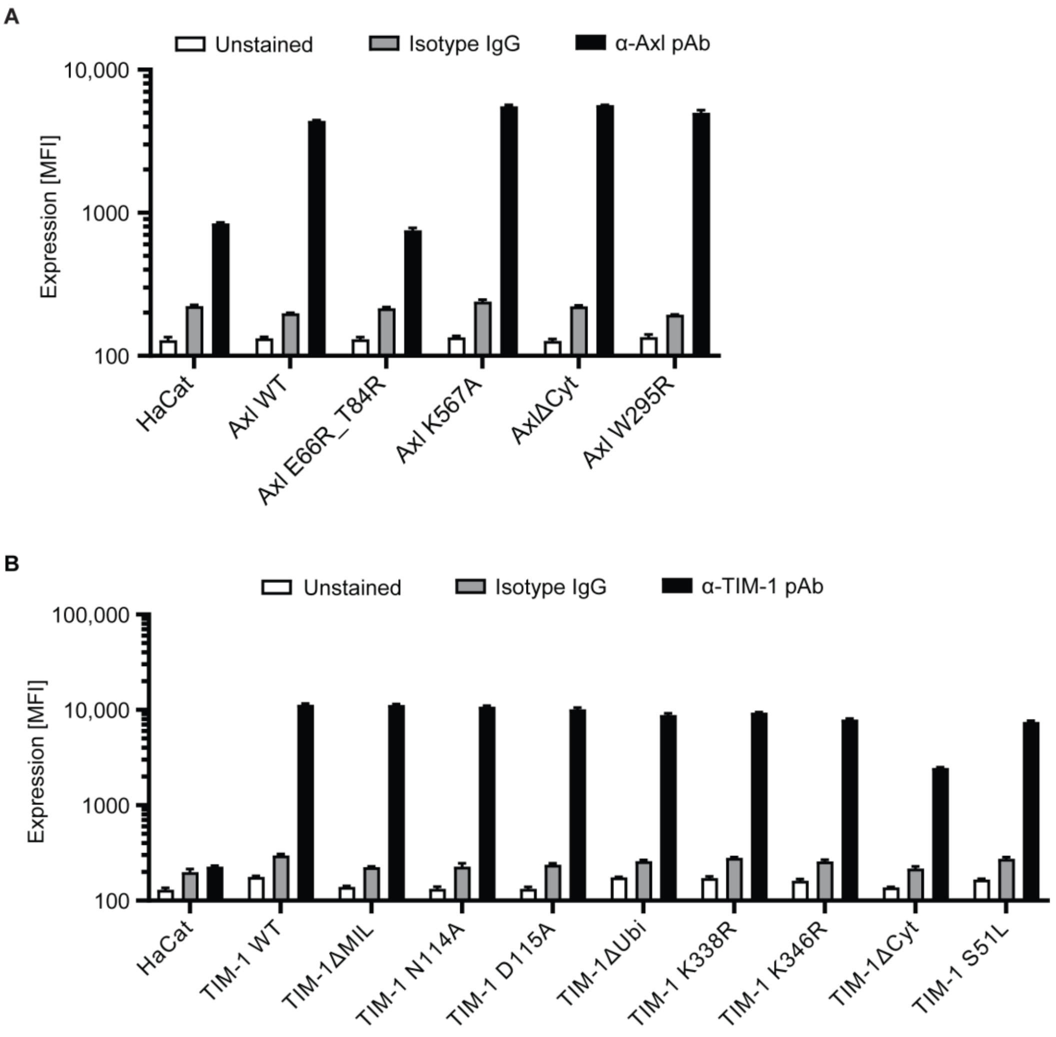

Figure S6. Expression of Axl and TIM-1 in HaCat cells. (**A**) Cell surface expression of Axl WT and Axl variants by HaCat cells analyzed by antibody staining and flow cytometry. (**B**) Cell surface expression of TIM-1 WT and TIM-1 variants by HaCat cells analyzed by antibody staining and flow cytometry. The error bars represent mean ± SEM of at least three representative replicates.

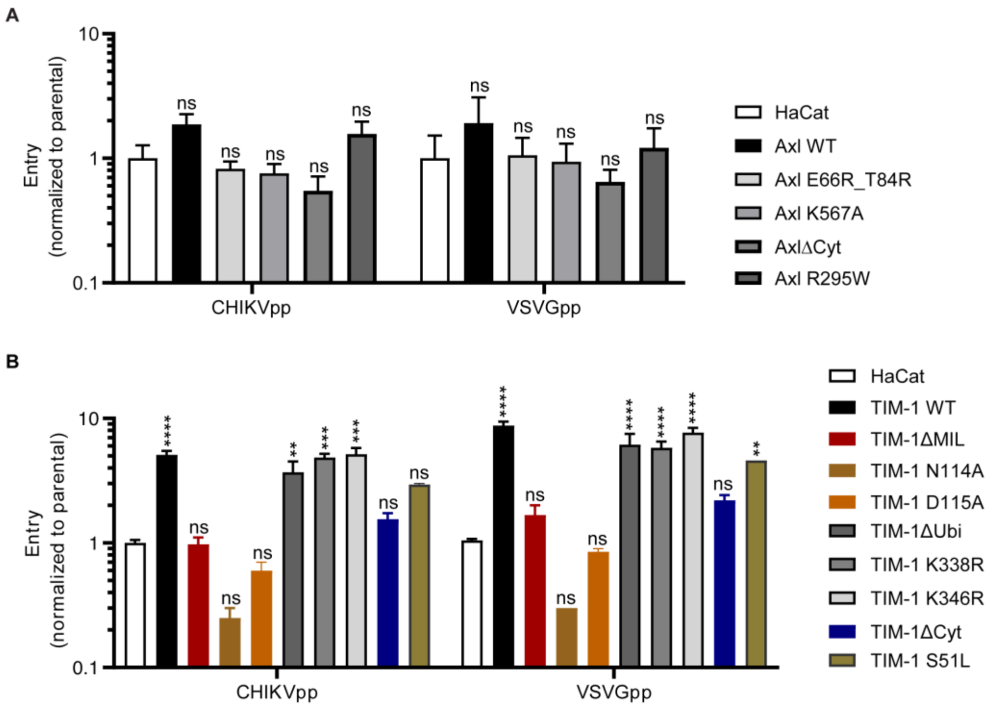

Figure S7. TIM-1 enhances CHIKV and VSV pseudoparticle entry in HaCat cells. Parental HaCat - immortalized keratinocytes and HaCat cells expressing; (**A**) Axl WT and variants (**B**) TIM-1 WT and variants were transduced with luciferase encoding lentiviral pseudoparticles-bearing glycoproteins of CHIKV or VSV. Entry was determined by luciferase assay and normalized to parental HaCat cells. The error bars represent mean ± SEM of at least three independent experiments. Statistical significance was calculated using a Dunnet's multiple comparisons test (2way ANOVA) $*p<0.05$, $**p<0.01$, $***p<0.001$ and $****p<0.0001$.

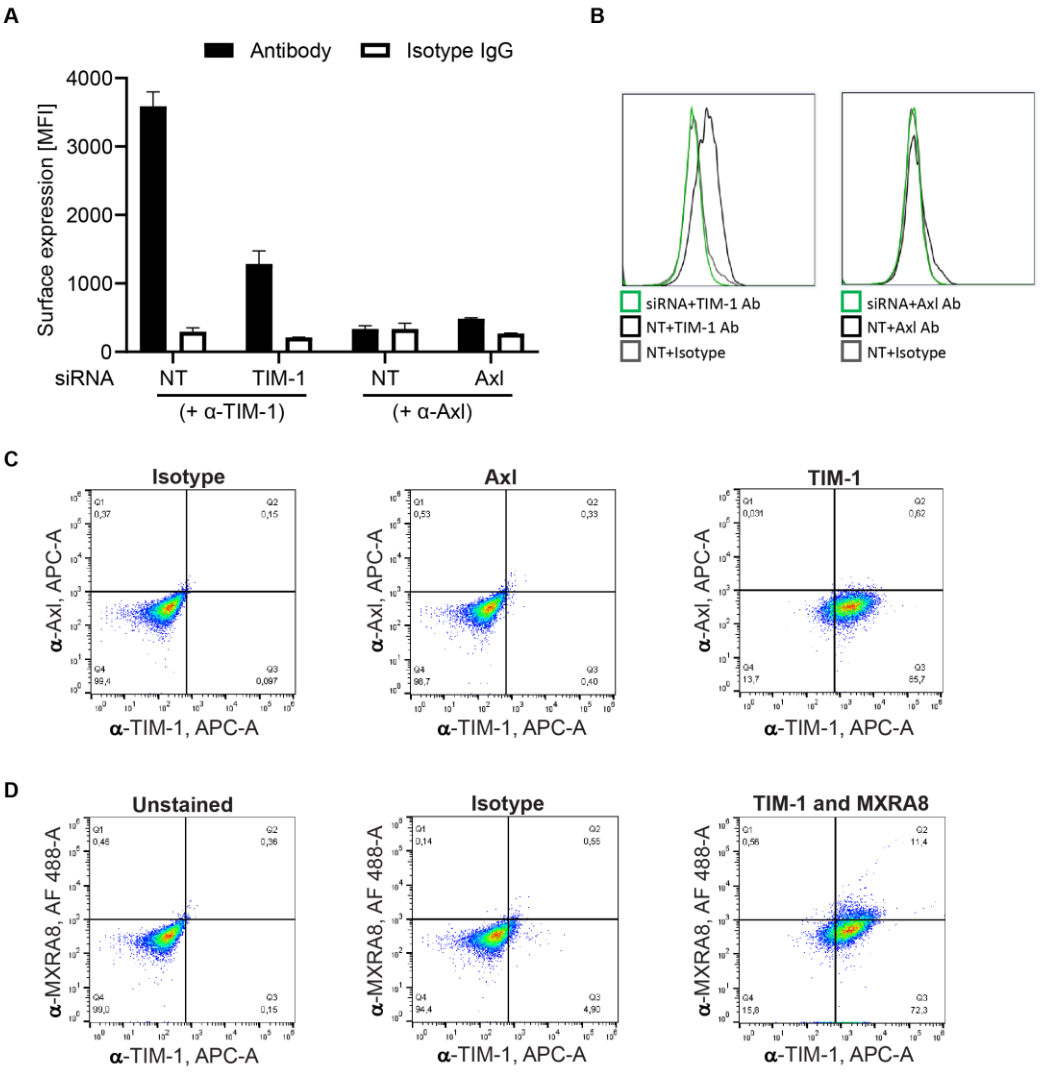

Figure S8: Surface expression of TIM-1, Axl and MXRA8 in Huh7.5 cells. (**A**) Graph showing expression level of TIM-1 and Axl in cells treated with either targeting or non-targeting (NT) siRNA. After 48 h the cells were stained with monoclonal antibodies for surface expression of TIM-1/Axl. (**B**) Histogram depicting the distribution of cells expressing TIM-1 or Axl. (**C**) Dot plot showing antibody control, Axl and TIM-1 staining (**D**) Dot plot showing unstained, antibody control, TIM-1 and MXRA8 staining of Huh7.5 cells expressing TIM-1 and/or MXRA8.

		nLuc-CHIKV (RLU)					
0 min	HEK293T	1521	2491	2867	2819	2812	2948
	TIM-1 WT	3884	4144	4301	4506	4527	4397
	TIM-1ΔMIL	2279	1992	2184	2737	2744	2648
	TIM-1ΔCyt	2737	2709	2839	4404	4520	4575
10 min	HEK293T	1508	1590	1583	3939	3912	4001
	TIM-1 WT	12596	12789	12686	4308	4281	4322
	TIM-1ΔMIL	1651	1644	1781	2566	2470	2470
	TIM-1ΔCyt	2778	2798	2703	4445	4513	4554
20 min	HEK293T	1392	1911	1535	1992	1945	1972
	TIM-1 WT	11162	27910	9311	4049	3823	3563
	TIM-1ΔMIL	1774	1549	1487	2286	2225	2013

	TIM-1ΔCyt	7010	8558	6230	2791	2675	2662
30 min	HEK293T	935	3386	2887	1945	1958	1958
	TIM-1 WT	27861	20962	20810	9202	11238	11101
	TIM-1ΔMIL	2614	2395	2245	2819	2880	2819
	TIM-1ΔCyt	17862	15330	13956	3659	3550	3700
60 min	HEK293T	6907	6401	6059	8092	9106	6545
	TIM-1 WT	125511	120343	104305	42200	49755	55290
	TIM-1ΔMIL	9880	10778	10456	11780	11437	11574
	TIM-1ΔCyt	60081	58162	57445	24495	26872	29266
120 min	HEK293T	23217	30118	23024	13626	10765	10909
	TIM-1 WT	417420	319246	309767	148887	142447	165227
	TIM-1ΔMIL	26402	26305	25427	25144	28857	26236
	TIM-1ΔCyt	240823	236166	255253	98093	89544	95671
240 min	HEK293T	22306	23155	23259	18585	16499	16651
	TIM-1 WT	644863	873281	639211	208850	181537	184888
	TIM-1ΔMIL	56659	45967	52101	109151	177960	118980
	TIM-1ΔCyt	414375	255245	264688	166552	97401	94489

Table S2: Raw data of the endosomal escape assay in relative light units (RLU). Parental HEK293T cells and cells expressing either TIM-1 WT or mutant variants were inoculated with nLuc-CHIKV and endosomal escape detected by luciferase assay.

Movies: Representative movies of mc-CHIKV diffusing at the surface of (**A**) TIM-1 WT and (**B**) mutant TIM-1ΔMIL. Both movies were recorded with an interval of 0.3 seconds between frames for 2 minutes. Videos were acquired 5 minutes after virus inoculum was added on cells (MOI=200). The trajectories of the landing viruses were reconstructed using TrackMate and are represented in yellow. Movies were processed in similar manner using Fiji: the time-lapse was smoothed followed by correction of uneven background with a rolling ball of 50 and the noise was despeckled twice. Finally, the LUT thresholds were set to be at a minimum of 150 and maximum of 500. The movies were saved with an acceleration of 50 frames per second.
